# Platelet concentrate in bovine reproduction: effects on *in vitro* embryo production and after intrauterine administration in repeat breeder cows

**DOI:** 10.1186/s12958-015-0064-6

**Published:** 2015-06-19

**Authors:** Anna Lange-Consiglio, Nadia Cazzaniga, Rosangela Garlappi, Chiara Spelta, Claudia Pollera, Claudia Perrini, Fausto Cremonesi

**Affiliations:** Large Animal Hospital, Reproduction Unit, Università degli Studi di Milano, Via dell’Università 6, 26900 Lodi, Italy; Private practitioner, 26833 Merlino Milan, Italy; Private practitioner, 27100 Pavia, Italy; Department of Veterinary Science and Public Health, Università degli Studi di Milano, 20133 Milan, Italy; Department of Veterinary Science for Animal Health, Production and Food Safety, Università degli Studi di Milano, 20133 Milan, Italy

**Keywords:** Repeat breeder cows, Embryos, Platelet concentrate, Intrauterine administration

## Abstract

**Background:**

A repeat breeder cow (RBC) can be defined as an animal that after 3 or more inseminations cannot get pregnant because of fertilization failure or early embryonic death. If no cause is identified precisely, inadequate uterine receptivity is responsible for implantation failures. Since a large number of identified molecular mediators, such as cytokines, growth factors and lipids have been postulated to be involved in early feto-maternal interaction, in this study a different approach to the treatment of RBC syndrome has been employed using a platelet concentrate (PC) that contains a significant amount of growth factors accumulated in its α-granules.

**Methods:**

Three explorative studies were performed. Initially, PC was supplemented in the *in vitro* embryo culture medium to study its effect on embryo-development. After the pilot study, 4 RBCs were treated with intrauterine administration of PC to evaluate proliferative potential of endometrium by immunohistochemical expression of the antigen Ki-67. Lastly, the effect of intrauterine administration of PC at 48 hrs after artificial insemination in RBCs was evaluated.

**Results:**

The *in vitro* results show that 5 % of PC and 5 % of fetal calf serum (FCS) increase the rate of blastocysts compared with the control containing 10 % FCS only (43.04 % vs 35.00 % respectively). The immunohistochemical study shows more proliferating nuclei in the treated uterine horn compared to the control one. After intrauterine insemination in RBCs, the percentage of pregnant cows in the control group was 33.33 % compared to 70 % of the treated animals.

**Conclusion:**

We suppose that when embryo descends in uterus could find a more appropriate environment for nesting and subsequent pregnancy.

## Background

Unexplained infertility, the so called repeat breeder cow (RBC) syndrome, involves a heterogeneous group of sub-fertile cows, with no anatomical abnormalities and infections of the reproductive tracts, nor estrous cycle alterations. A repeat breeder is defined as a cow that fails to become pregnant after 3 or more inseminations as a consequence of either fertilization failure or early embryonic death [1].

The etiology of RBC syndrome is multi-factorial and unclear. The normal uterine environment promotes normal embryonic development but subclinical disorders compromise the survival of the embryo resulting in RBC syndrome. The RBCs are characterized by early embryonic loss [2], that decreases the conception rates [3, 4]. In some cases, the transfer of non-RBC donated embryos into RBC surrogates increased RBC pregnancy rate, indicating that the lower conception rate of RBCs is determined at early stages of embryo development [3]. The problem could be the embryo but also the oviductal and/or uterine environment. Ferreira *et al.* [5] provided evidence that RBC syndrome is associated with oocyte quality and that this negative effect is enhanced during summer heat stress, but it is general opinion that the successful implantation requires also a complex sequence of signaling events that are crucial to the establishment of pregnancy. In human medicine there is a proportion of women with ‘unexplained’ infertility in whom pregnancies fail before they are clinically recognized. Koot *et al.* [6] underline that this infertility could occur as a result of a malfunction of the endometrial-embryo ‘dialogue’ after the early phases of implantation. Indeed, the uterus is responsible for two-thirds of failures whilst the embryo for only one-third [7, 8]. A large number of molecular mediators, under the influence of ovarian hormones, have been postulated to be involved in this early embryo-maternal interaction. These mediators embrace a large number of inter-related molecules including adhesion molecules, cytokines, growth factors, lipids and others [9, 10]. Many treatments have been proposed for prevention of RBC syndrome at both herd and individual level. These include, for example, nutritional supplements and assisted reproductive techniques, such as *in vitro* embryo production and embryo transfer. Commonly, therapies in use include hormonal treatments with progestins, GnRH, exogenous gonadotrophins and prostaglandins [1]. However, in view of the embryo-maternal interaction a different approach to the treatment of RBC syndrome could be found using platelet concentrate (PC). Platelets contain significant quantities of growth factors (accumulated in their α-granules), chemokines and cytokines and also active metabolites [11], that act in a paracrine manner on different cell types like myocytes [12], mesenchymal stem cells of different sources [13], condrocytes [14, 15], osteoblasts [16], fibroblasts [17]. Moreover, several *in vitro* studies have shown a direct dose–response influence of many growth factors on cell migration, cell proliferation, and matrix synthesis [18–20]. Transforming growth factor β1 (TGF-β1) and TGF-β2, platelet derived growth factors (PDGF-AA, PDGF-BB, PDGF-AB), insulin-like growth factor 1 (IGF-I), epidermal growth factor (EGF), vascular endothelial growth factor (VEGF), fibroblast growth factor (FGF) and hepatocyte growth factor (HGF) are very important for regeneration processes. Indeed, these growth factors act synergistically to increase the infiltration of neutrophils and macrophages, to promote angiogenesis, fibroplasia, matrix deposition and, ultimately, re-epithelialization, inducing the consequent tissue regeneration [21]. Lastly, it is known the anti-inflammatory property of PC by the presence of anti-inflammatory agents including HGF [22].

In this context, the uterine administration of PC may be useful in peri-implantation, or in the healing process of clinically silent endometrial injuries because many cytokines act as intermediary links in the materno-fetal relationship including decidualization (in the women), implantation, placentation, embryogenesis and fetal growth [23]. Moreover, since pro-inflammatory factor transcripts in bovine endometrial epithelial cells are elevated in case of subclinical or clinical endometritis [24], we hypothesized that an early administration of PC, after artificial insemination (AI) and before the descent of the blastocyst in the uterus, could improve the uterine microenvironment for embryo implantation and counteract eventual subclinical endometritis.

## Methods

### Materials

Chemicals were obtained from Sigma Chemical (Milan, Italy) and tissue culture plastic dishes from Euroclone (Milan, Italy) unless otherwise specified.

### Experimental Design

This study was based on three experiments as summarized in Table 1. The first experiment was to evaluate the effect of PC on *in vitro* embryo production by replacing fetal calf serum (FCS) with PC to establish whether this product is able to support embryo development. The second experiment evaluated the endometrium immunohistochemically, after *in vivo* PC administration, using Ki-67 as a marker of cell proliferation. The third *in vivo* experiment evaluated embryo implantation and development in RBCs following intrauterine administration of PC at 48 h after artificial insemination (AI).

**Table 1:** Experimental design

Experiments		N° samples	Parameter evaluated
1	*In vitro*: effect of different amounts of PC on embryo development	705 oocytes	• Rate of embryos • Total cell numbers per blastocyst
2	*In vivo*: evaluation of endometrial cell proliferation, after *in vivo* PC administration	4 RBCs	Microscopic nuclear count of cells expressing Ki-67
3	*In vivo*: embryo implantation and development after intrauterine administration of PC	30 Treated RBCs 30 Control RBCs	Rate of pregnancy

### Animals and repeat breeder syndrome diagnosis

All procedures were performed according to approved animal care and use protocols of the institutional ethics committee and to good veterinary practice for animal welfare as to European directive 2010/63/UE. Moreover, written farmers’ consent was obtained at the beginning of the study.

The study was performed between January and April to reduce the influence of climate (*i.e.* a hot and humid climate, heavy rain, heat stress). Indeed, in the Italian environment late winter is probably the most stable period, with a low rain rate and temperature between 0 and 10 °C. A total of 64 animals Holstein Friesians in which pregnancy had not been achieved despite 3 or more inseminations were enrolled in this study. Ultrasound examination was performed in each cow to detect any reproductive pathology (like ovarian cysts, pyometra, abscess…) that would result in exclusion of cows from the experiment because not considered RBCs. For each cow, on the day of the estrus, cervical mucus was tested and samples of cervical mucus were collected aseptically by swab (Equi-Vet, Kruuse, Marslev, Denmark). Bacteriological examinations were performed as reported by Gani *et al.* [25].

The RBCs were 385 ± 12.5 days in milking, had a weekly milk production of 128.6 ± 0.7 kg, 6.05 ± 4.84 previous inseminations (minimum of 3 and maximum of 23 inseminations per animal), and were on lactation number 2.7 ± 0.2.

### Preparation of Platelet Concentrate (PC)

Blood was obtained from 8 donor cows, at the forty days in milking because in this period the circulating platelet counts is higher than other periods (data not shown), in good health, free from infection that had not received medication during the previous two months. The collection of blood and the preparation of PC, with the method of double centrifugation, were performed as reported by Lange-Consiglio *et al.* [26]. Briefly, after surgical scrub preparation of a few centimetres of skin around the subcutaneous mammary vein, 450 ml of blood was collected in *ad hoc* Terumo blood bags (Terumo Srl, Rome, Italy) containing CPDA-1 by using the 16 gauge needle provided with the bags. The bags were transported at +4 °C to the laboratory within 2 hrs of collection and immediately processed. All separation steps to produce PC were performed under a horizontal laminar flow hood in aseptic conditions. To prepare the PC, the blood was drawn into sterile Falcon tubes of 50 ml each. The tubes were centrifuged at 100xg for 30 min. This caused separation of the blood into three components: red blood cells at the lowest level, ‘buffy coat’ comprised in the middle layer, and platelet-rich plasma (PRP) in the upper layer. Afterward, the PRP was carefully aspirated and distributed in new 50 ml tubes and centrifuged again at 1500xg for 10 min to obtain the platelet pellet and the poor platelet plasma (PPP) at the upper layer. Afterwards, two thirds of the volume of PPP was aspirated for later use and the pellet mixed in the residual PPP volume to allow for platelet count before the final dilution with PPP to obtain PC at a standard concentration of 1x10^9^ platelet/ml. All platelet counts on peripheral blood, PRP and PC were performed by an automatic hematology analyser HeCo Vet SEAC (Florence, Italy).

At the end of the process, PC was pooled and prepared at a standard concentration of 1 × 10^9^ platelet/ml. The total volume of PC was aliquoted into 10 ml usage doses that were stored in syringes. To release platelet derived factors, three cycles of freezing at −80 °C and thawing at 37 °C were performed [27]. Syringes containing PC dose were kept frozen at −20 °C until use. The same pool of PC obtained from donor cows was used in all experiments and in all animals in an heterologous way meaning that it was employed in cows different from donor cows.

For supplementation in *in vitro* cultured embryos, to prevent coagulation and clot formation, a fibrinogen free PC was prepared as reported by Mojica-Henshaw *et al.* [28]. Briefly, after thawing, pooled PC was centrifuged at 4000 g for 20 min, and the supernatant was collected and was manufactured by adding calcium chloride (20 % w/v) at a ratio of 1:100. After allowing the product to form a clot overnight at 4 °C, the coagulated product was centrifuged at 4000 g for 20 min, and the supernatant was collected to obtain fibrinogen free PC.

### Experiment 1: effect of PC on *in vitro* embryo production

The *in vitro* embryo production consisted of 3 steps: *in vitro* maturation of oocytes (IVM), *in vitro* fertilization (IVF) and *in vitro* culture of embryo (IVC) performed on a monolayer of cumulus cells. These steps were carried out as reported by Lange-Consiglio *et al.* [29]. Briefly, cumulus–oocyte complexes (COCs) were collected from ovaries obtained from an abattoir by aspirating follicles 2–8 mm in diameter and washing them twice in preincubated (38.5 °C, 5 % CO_2_ in air) TCM 199-HEPES buffered culture medium supplemented with 10 % FCS.

IVM was performed for 24 h in TCM 199 Earl’s Salt medium supplemented with 10 % FCS, 5 μg/ml LH (Lutropin, Bioniche, Canada), 0.5 μg/ml FSH (Folltropin, Bioniche, Canada), 0.2 mM sodium pyruvate, 10 μg/ml gentamycin and 1 mg/ml estradiol 17β. Cultures were in 70 μl droplets (up to 20 oocytes/droplet) of the medium under oil, at 38.5 °C in 5 % CO_2_.

IVF was performed in Tyrode’s-albumin-lactate-pyruvate (TALP) medium containing 2 mM penicillamine, 1 mM hypotaurine, 250 mM epinephrine and 20 μg/ml heparin. Frozen–thawed semen was prepared by Percoll gradient (Amersham Pharmacia Biotec, Upsala, Sweden). In a 15 ml conic tube, 1 ml Percoll 90 % was added followed by 1 ml Percoll 45 %. Semen was thawed at 37 ° C for 30 sec, placed on the top of the Percoll gradient and centrifuged for 30 min at 300 xg. After removal of the supernatant, 4 ml TALP medium were added and the sample centrifuged again for 2 min at 200 xg to remove excess Percoll.

Semen (10^7^ spermatozoa/ml) was co-incubated with matured oocytes for 18 h at 38.5 °C in 5 % CO_2_. At the end of gametes co-culture, the cumulus cells were completely removed and evaluation of segmentation was carried out from the first day of embryo culture (considering as day zero the day of insemination) when embryos were equally divided into three different embryo culture media: 1) standard embryo culture medium (TCM-199) with 10 % of FCS; 2) standard medium supplemented with 5 % FCS and 5 % PC; 3) standard medium supplemented with 10 % PC. The percentage of embryos developing to blastocyst stage was evaluated daily up to day 7 after fertilization. Some blastocysts, as reported in Table 2, were stained with Hoechst 33342 (10 μg/ml) for 10 min, and total cell numbers were counted under an epifluorescence microscope to estimate embryo quality. Hoechst 33342 dye was excited at 353–365 nm while the emission wavelength was set at 460 nm.

### Experiment 2: immunohistochemical analysis of endometrium after *in vivo* PC administration

Four RBCs, belonging to the group previously described, culled by owner decision were used to test the effect of PC on endometrial cell proliferation. On the ninth day of diestrous stage, 10 ml of PC were administered into uterine horn ipsy-lateral (two cows) or contra-lateral (two cows) to the corpus luteum, maintaining the opposite horn as a control. After PC administration, a Foley catheter was inserted in the control horn and the balloon was inflated to prevent passage of PC and create the control environment. Our previous study (data not shown) demonstrated that insertion of a Foley catheter for 3 days does not alter the normal histological appearance of endometrium. These cows were slaughtered 3 days after the treatment (on the twelfth day of diestrus stage) and the 4 uteri were evaluated macroscopically for differences between the treated and the control horns. For each horn, samples of middle/cranial portion were collected in formalin solution and were examined immunohistochemically for expression of the nuclear antigen Ki-67, a marker of cell proliferation, using the ABC-peroxidase method as reported by Turner *et al.* [30]. Briefly, the tissue was fixed in 4 % buffered formalin, dehydrated, and embedded in paraffin. Sections 4 μm thick were mounted on aptes (3-aminopropyl triethoxy silane, Sigma) coated slides, dewaxed, and rehydrated. Sections were immunostained using the standard ABC technique with the rabbit antihuman/horse polyclonal antibody to Ki-67 (Dako Italia, S.p.A., Milan. Italy) used at 1:50 dilution. Ki-67 antibody is directed against different epitopes of the proliferation-related antigen and can be used on fixed sections. Endogenous peroxidase activity was blocked using 3 % hydrogen peroxide. Pre-treatment with microwaving in sodium citrate buffer, pH 6, was used. Non-specific primary antibody binding was blocked using fetal calf serum at a dilution of 1:20 for the ABC Ki-67. The primary antibody was applied for 18 hrs at +4 °C and washed in buffered saline. An antirabbit biotinylated secondary antibody (Insight Biotechnology Limited, Wembley Middlesex, UK) was applied at 1:200 dilution for 30 min at room temperature, followed by washes and then by application of the horseradish peroxidase streptavidin complex (Dako) at 1:400 dilution for 30 min. The anti-rabbit secondary antibody is an affinity-purified polyclonal antibody with well-characterized specificity for rabbit IgG. Colour development was with metal enhanced diaminobenzidine (DAB) (Pierce and Warriner Ltd, Chester, UK) applied for 15 min. The slides were lightly counterstained with Mayer emallume. The sections were viewed under a photomicroscope Olympus BX51 and nuclear count was performed on each section, at the level of endometrial epithelial cells, as number of positive cells to detect the entity of cell proliferation. Three different sections per cow and 4 microscopy fields for each section were counted.

### Experiment 3: *in vivo* effectiveness of intrauterine administration of PC at 48 h after AI in RBCs

This experiment was carried out in 2 farms, managed in a cubicle yard. Sixty RBCs were randomly allocated into two groups: “treated group” (N = 30) that received intrauterine administration of PC 48 hrs after AI and “control group” (N = 30), that received no treatment in addition to AI. Treated group were characterized by 6.0 ± 5.19 inseminations prior to the enrollment in the study while control cows showed 6.1 ± 4.49 inseminations.

Cows displaying estrus in either group were submitted for insemination. The semen from the same bull was used for insemination in each group, eliminating the “bull” effect in the results of fertility.

All the cows were blood sampled for progesterone assay at day 0 (day of insemination), and then 4 and 8 days after insemination (respectively T0, T4 and T8). At 48 hrs after insemination, 10 ml of PC at 1 x 10^9^ platelet/ml were administered into the uterus in the cows in the treated group. This time was supposed to be ideal after AI not to disturb spermatozoa progression and before the embryo reaches the uterus. Ten ml of PC were administrated following previous study (data not shown) in which this volume proved to be evenly distributed along the uterine horns without signs of excessive distension allowing to reach the deepest portion of the organ. Ultrasound examination for pregnancy diagnosis was performed in all cows 32 days after insemination and confirmed at 60 days.

### Bacteriological examination of cervical mucus

Bacteriological investigations were performed to detect aerobic and anaerobic microorganisms, such as *Trueperella pyogenes, Haemophylus sommni, Staphylococcus sp., Streptococcus sp., Enterobacteriaceae*, *Prevotella sp., Fusobacterium spp*. Each sample was plated on blood agar (agar with 5 % sheep blood, Sintak, Italy) for isolated colonies. The plates were incubated for 24–48 hrs in aerobic and anaerobic conditions at 37° ± 2 °C. Identification of bacteria was based on colonial morphology, Gram staining, and biochemical tests such as catalase, oxidase, coagulase according to the guidelines of the Bergey’s manual of Systematic Bacteriology and the standard bacterial procedure of CLSI (Clinical and Laboratory Standards Institute) and confirmed by API-System (bioMerieux, France).

### Intrauterine administration of platelet concentrate

After thawing and warming at 38 °C, the syringes of PC were connected to a sterile disposable intrauterine cannula, so that samples could be delivered through the vestibule, the vagina and the cervix to reach deep into the uterine horn ipsi-lateral to the newly formed corpus luteum. Plastic catheters with atraumatic ends were used. Ten ml of PC were administrated to cows in the treatment group 48 hrs after insemination.

### Progesterone assay

Plasma samples were obtained by jugular venipuncture (2-5 ml). Blood was collected in heparinized tubes and centrifuged at 1000xg for 10 min. The plasma concentration of progesterone was assessed using a quantitative automated method based on the enzyme-linked fluorescent assay (ELFA) technique (Mini-Vidas; bioMérieux Italia S.p.A., Florence, Italy.

### Statistical analysis

The collection efficiency of platelets for each PC was analyzed using the following formulas [31]:

$$ \mathrm{efficiency}\kern0.5em \mathrm{f}\mathrm{o}\mathrm{r}\kern0.5em \mathrm{platelet}\kern0.5em \mathrm{collection}=\frac{\mathrm{platelet}\kern0.5em \mathrm{count}/\upmu \mathrm{l}\kern0.5em \mathrm{in}\kern0.5em \mathrm{P}\mathrm{C}\kern0.5em \mathrm{x}\kern0.5em \mathrm{volume}\kern0.5em \mathrm{o}\mathrm{f}\kern0.5em \mathrm{P}\mathrm{C}}{\mathrm{platelet}\kern0.5em \mathrm{count}\kern0.5em \mathrm{in}\kern0.5em \mathrm{whole}\kern0.5em \mathrm{blood}/\upmu \mathrm{l}\kern0.5em \mathrm{x}\kern0.5em \mathrm{volume}\kern0.5em \mathrm{o}\mathrm{f}\kern0.5em \mathrm{whole}\kern0.5em \mathrm{blood}} $$

The statistical analysis of the data collected during the study, except for bacteriological data, was conducted using chi-square test. For all tests, differences were considered statistically significant when *P* ≤ 0.05. The data were analyzed using the “Software GraphPad Instat 3.00 for Windows” (La Jolla, CA, USA).

## Results

### Platelet collection efficiency and product sterility test

The platelet collection efficiency for the PCs was 10.9 ± 2.3 %, indicating that 10.9 ± 2.3 ml of PC at the concentration of 1x10^9^ platelet/ml were obtained from 100 ml of blood.

The bacterial sterility test performed on the PC produced in the laboratory has always given negative results, confirming that the experimentation has been performed in accordance with the appropriate standards of laboratory sterility.

### Experiment 1: effect of PC on *in vitro* embryo production

During three experiments, 168 ovaries were processed, 705 oocytes were collected, with an average of 4.2 oocytes collected per ovary. The results related to the culture of embryos in the standard medium (CTR) show that the rate of cleavage of three replicates is 66.80 ± 2.07.

Results related to embryo culture show that the rate of blastocyst development was statistically significant different (*P* < 0.001) among all the three media tested and the best embryo production rate and quality was achieved in the culture medium supplemented with 5 % PC and 5 % FCS (Table 2).

**Table 2:** Number of blastocysts at 7 days in three different culture media and total cell number per blastocysts

Medium	N° of blastocysts at 7 days (%)	N° of embryos observed	Total cell numbers per blastocyst
CTR (10 % FCS)	84/240 (35.00 ± 1.72)^a^	29	104.6 ± 10.5^a^
FCS 5 % + PC 5 %	99/230 (43.04 ± 1.42)^b^	31	123.4 ± 8.3^b^
PC 10 %	64/235 (27.23 ± 1.39)^c^	34	98.7 ± 6.4^c^

The number are expressed as mean ± standard deviation (SD)

Different small letters superscript (a, b) in the same column indicate statistically different comparisons (*P* < 0.05)

### Experiment 2: immunohistochemical analysis of endometrium after *in vivo* PC administration

**Fig. 1:**
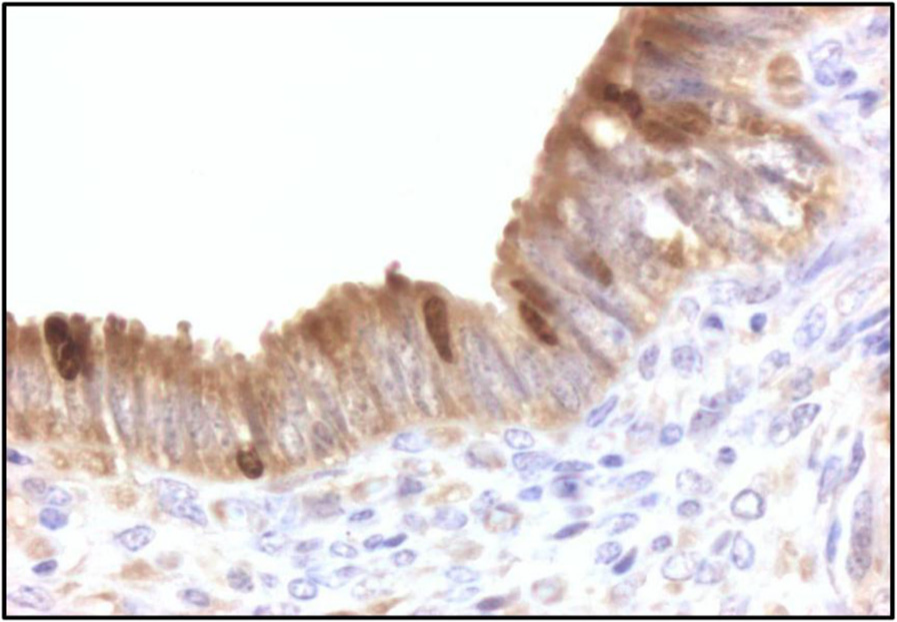
Immunohystochemical analysis. Nuclear positivity to Ki-67 protein. Magnification 100x

No macroscopic alterations were observed in any of the uterine samples. Immunohistochemical examination for the unmasking of the nuclear antigen Ki-67 showed that in all animals there were statistically more proliferating nuclei in the treated uterine horn compared to the control one (Table 3 and Fig. 1).

**Table 3:** Microscopic nuclear count of cells expressing nuclear antigen Ki-67 protein in control (CTR) and treated (TRT) uterine horns of 4 cows in different sections and different microscopic fields

1		2		3		4	
Uterine horn		Uterine horn		Uterine horn		Uterine horn	
CTR	TRT	CTR	TRT	CTR	TRT	CTR	TRT
2 ± 0.7^a^	12 ± 0.2^b^	1 ± 0.2^a^	10 ± 0.6^b^	5 ± 2.9^a^	18 ± 2.3^b^	14 ± 3.8^a^	40 ± 4.2^b^

Legend: CTR Control; TRT Treated. The number are expressed as mean ± standard deviation (SD)

Different small letters superscript (a, b) in the same line indicate statistically different comparisons (*P* < 0.05)

### Experiment 3: *in vivo* effectiveness of intrauterine administration of PC at 48 hrs after AI in RBCs

The clinical aspect of the uterus in all animals during the clinical examination at the estrous stage appeared normal in size, without presence of drains and with adequate, clear and stringy estrous mucus.

Progesterone values did not differ from those expected in a normal estrous cycle. Indeed, these values were lower than 1 ng/ml during peri-estrus and then increased gradually at day +4 as a result of ovulation and further increased at day +8 post insemination (Table 4). The percentage of pregnant cows in the control group was of 33.33 % (10/30) compared to 70 % (21/30) of the treated animals. The difference in pregnancy rates between the treated group and the control group was statistically significant (*P* < 0.05; Table 4). There was no correlation between treatment results and number of past inseminations before this study and characterizing the status of the enrolled RBCs (*P* = 0.62). All treated and control RBCs diagnosed as pregnant gave birth to normal offsprings.

**Table 4:** Results of *in vivo* effectiveness of intrauterine administration of PC 48 h after AI in RBCs

		Progesterone value (ng/ml)					
	N°cows	T0	T4	T8	Pregnant (%)	Not Pregnant (%)	Delivery (%)
Control	30	0.40 ± 0.21^a^	2.63 ± 0.96^a^	7.75 ± 2.21^a^	10 (33.33)^a^	20 (66.67)^a^	10 (100)
Treated	30	0.40 ± 0.17^a^	3.12 ± 0.85^a^	8.33 ± 2.14^a^	21 (70.00)^b^	9 (30.00)^b^	21 (100)

Different small letters superscript (a, b) in the same column indicate statistically different comparisons (*P* < 0.05)

### Microbiological results

No significant bacterial growth was observed in any of the cervical mucus samples obtained from cows of both experimental groups. The microorganism *Aerococcus viridans* was isolate only from one cow.

## Discussion

Studies in humans and in other mammals have shown that cytokines and growth factors are produced by the pre-implantation embryo and cells of the reproductive tract, even if the exact combinations required for successful implantation are not yet fully understood [23]. Based on the current knowledge of the regenerative effect of PC due to its high growth factor and cytokine content [22], this study proposes a novel therapeutic approach. Autologous PC is known to accelerate the healing process in human medicine, and has been used in maxillofacial surgery, muscle and/or tendon repair, and reversal of skin ulcers [21]. In veterinary medicine there are few clinical reports of the use of PC. It has been mainly used for promoting equine tendon repair [32], but there are some reports of its use in intestinal wound healing in pigs [33], in a large cutaneous lesion in a dog [34] and in bovine mastitis in which it was used in heterologous way [26]. In all cases PC or platelet rich plasma showed a clear regenerative potential.

For this reason, in this preliminary investigation, we performed three explorative studies *in vitro* and *in vivo*. Only one paper [35] described the effects of platelet rich plasma on morula and blastocysts *in vitro* production. In our study and in our experimental conditions (*i.e.* platelet concentration at 1x 10^9^/ml), at first we studied the effect of PC on *in vitro* embryo development using two different percentages (respectively 5 % and 10 %) of PC as a partial or complete replacement of FCS. The rate of blastocyst production and the total cells number of the blastocysts were statistically increased in the medium with 5 % PC and 5 % FCS when compared to both the control and medium with 10 % PC. Platelets release many growth factors, including FGF, TGF-6, PDGF and EGF [36, 37] that can stimulate bovine embryo development [38, 39]. Indeed, Munson *et al.* [40] demonstrated that TGF-6 and PDGF act synergistically to promote proliferation of both bovine trophoblastic cells and endometrial epithelial cells during *in vitro* culture. Moreover, EGF *in vivo* is produced by endometrial cells and its receptors have been detected in the embryo itself, where EGF acts through the phosphorylation of membrane proteins as a mitogen, promoting DNA and RNA synthesis. As pregnancy progresses, the increased production of EGF enhances trophoblast differentiation [41], promoting cell attachment and embryo development [23]. Fibronectin and other glycoproteins are also released from platelets after aggregation, and Larson *et al.* [42] discovered that a serum-free medium supplemented with fibronectin provides the extracellular matrix required by the embryo to develop to the blastocyst stage during *in vitro* culture. In a speculative interpretation, the embryo culture systems supplemented with PC may have provided TGF-6, PDGF and the matrix of extracellular fibronectin necessary to support the development of embryo during *in vitro* culture, thus replicating the uterine microenvironment appropriate for embryo growth, viability, and for cytokine secretion [23]. It is possible that the low rate of embryos obtained in the medium with 10 % PC, compared to control, results from an excess of factors that may have had an inhibitory effect on the embryo development.

This *in vitro* study provided a useful starting point for the pre-clinical investigation in order to use PC in *in vivo* trials. Immunohistochemical examination of uterine samples from animals slaughtered 3 days after PC treatment showed that there was more expression of the nuclear antigen Ki-67 in samples from treated uterine horns compared to the controls. Cell proliferation is not enough indicative for uterine health but Ki-67 is expressed in the nuclei of proliferating cells. In tissues with a high proliferative rate it is expressed in all cell cycle phases except for the resting or G0 phases [43, 44] and reaches a maximum level during G2 and M [45]. Since Ki-67 is significantly more expressed in samples from treated uterine horns, it may be supposed that the growth factors released by platelets in the PC may have an effect on endometrial cell proliferation.

After these preliminary studies, the *in vivo* administration of PC into the uterus of RBCs at 48 hrs after insemination was performed.

The gynecological clinical status and the progesterone analysis performed in this study ensured that pregnancy losses in enrolled animals were not caused by abnormal ovarian cycles, an important condition for classification of cows as RBC, as reported by Gustafsson *et al.* [46]. The progesterone analysis also allowed us to exclude hypoluteinism as a possible cause of repeat breeding. In fact, all animals showed progesterone values exceeding the 2 ng/ml threshold both at day +4 and +8 post-insemination [47]. Moreover, bacteriological examination performed on cervical mucus confirmed the absence of bacterial infections in the genital tract, as the only isolated microorganism was *Aerococcus viridans,* a bacterium not recognized as a uterine pathogen.

In the *in vivo* study, there were more pregnant cows in the treated group compared to the control group. This suggests that the *in vivo* intrauterine administration of PC 48 hrs after insemination, time supposed to be ideal after AI not to disturb spermatozoa progression and before the embryo reaches the uterus, could make the uterine environment favorable to embryo implantation. This may be the effect of platelet growth factors on regeneration and/or the healing of clinically silent endometrial abnormalities. Furthermore, PC should enrich the uterine environment with factors necessary for embryo development. Uterine glands produce a glycoprotein-rich secretion, which is believed to support the embryo during the pre-implantation period [48]. In addition to providing nutrition for the conceptus, this secretion contains a complex array of growth factors and cytokines. Katagiri and Takahashi [49] reported that RBCs show abnormalities in the growth factor-cytokine network - specifically in endometrial EGF concentrations. Cyclic cows have two peaks of EGF concentrations on days 2–4 and 13–14 of the oestrous cycle, but these peaks are lost or diminished in about 70 % of RBCs [49]. The EGF family could act on the trophectoderm promoting cell attachment and embryo development [23]. Since PC contains EGF, treatments targeting the endometrial growth factor regulatory network may be an effective way to deal with RBCs when uterine problems limit the production of this or other growth factors.

## Conclusions

The preliminary data regarding the administration of PC into the uterus of animals at 48 hrs after insemination produced very encouraging results in RBCs. From our data, we hypothesize that PC, whose content is known, enriches the uterine environment with factors necessary for embryo development and counteracts eventual subclinical endometritis by its anti-inflammatory properties. The obtained results in our three different explorative studies are very interesting. This research could offer a new therapeutic strategy for RBC syndrome and also open the possibility for using PC in future embryo-transfer programs in both human and veterinary medicine as a vehicle to transfer embryos at the time of transplantation, with the aim of improving the uterine environment for implantation.

## Abbreviations

AI: artificial insemination
EGF: Epidermal Growth Factor
FCS: Fetal Calf Serum
FGF: Fibroblast Growth Factor
HGF: Hepatocyte Growth Factor
IGF-I: Insulin-like Growth Factor 1
IVC: *in vitro* Culture of Embryo
IVF: *in vitro* Fertilization
IVM: *in vitro* Maturation
PC: Platelet Concentrate
PDGF-AA: PDGF-BB, PDGF-AB, Platelet Derived Growth Factors
RBC: Repeat Breeder Cow
TGF-β1: Transforming Growth Factor β1
TGF-β2: Transforming Growth Factor β2
VEGF: Vascular Endothelial Growth Factor
